# Nanocellulose: Recent advances and its prospects in environmental remediation

**DOI:** 10.3762/bjnano.9.232

**Published:** 2018-09-19

**Authors:** Katrina Pui Yee Shak, Yean Ling Pang, Shee Keat Mah

**Affiliations:** 1 Department of Chemical Engineering, Lee Kong Chian Faculty of Engineering and Science, Universiti Tunku Abdul Rahman, Jalan Sungai Long, Bandar Sungai Long, Cheras 43000 Kajang, Selangor Darul Ehsan, Malaysia

**Keywords:** adsorbent, environmental remediation, membrane, nanocellulose, nanomaterials, photocatalyst

## Abstract

Among many other sustainable functional nanomaterials, nanocellulose is drawing increasing interest for use in environmental remediation technologies due to its numerous unique properties and functionalities. Nanocellulose is usually derived from the disintegration of naturally occurring polymers or produced by the action of bacteria. In this review, some invigorating perspectives on the challenges, future direction, and updates on the most relevant uses of nanocellulose in environmental remediation are discussed. The reported applications and properties of nanocellulose as an adsorbent, photocatalyst, flocculant, and membrane are reviewed in particular. However, additional effort will be required to implement and commercialize nanocellulose as a viable nanomaterial for remediation technologies. In this regard, the main challenges and limitations in working with nanocellulose-based materials are identified in an effort to improve the development and efficient use of nanocellulose in environmental remediation.

## Review

### Introduction

The rampant emergence of advanced, new nanomaterials is redefining many industries and blurring traditional research boundaries. In the drive towards sustainable development, the growing application of renewable, biodegradable, green materials to substitute non-renewable resources have roused substantial interest on a global scale. In addition, the uprising environmental concerns warrant the application of bio-renewable polymers in the production of nanomaterials.

In recent years, nanomaterials have displayed potential in effective detection and removal of greenhouse gases, chemical contaminants, organic pollutants, and biological agents. These materials come in various morphologies and have various functions (e.g., adsorbents, catalysts, or membranes). The high reactivity and high surface area of nanomaterials are some of the notable features which provide an advantage in environmental remediation over other conventional alternatives [1]. Promising materials such as graphitic carbon nitride [2–3], carbon nanodots [4], and two-dimensional carbon-based nanocomposites [5–7] are a few trending nanomaterials that have already found extensive applications in both environmental remediation and energy generation. In the past, carbon nanotubes (CNTs) have received a great deal of attention as materials for environmental remediation due to their impressive mechanical properties and superior adsorption capability. However, the need for non-renewable sources such as fossil fuel to produce CNTs remains a challenge. As of late, research progression in environmental science continues to push for materials which are renewable, biocompatible, and less toxic as a replacement for CNTs.

Given the abundance of plant resources, plant extracts are the most studied category to date for the synthesis of green nanomaterials [8]. Cellulose, one of the most abundant natural polymers, has the potential to overcome challenges pertaining to material biodegradability, renewability, cost, and energy. Furthermore, cellulose found in most lignocellulosic biomass, derived from agricultural waste, energy crops and forestry residues, provides a better option over food crops as a renewable resource for the production of new materials due to its availability in large amounts and low cost [9]. Moreover, cellulose is known as a nontoxic material [10–12].

In the past few decades, nanocellulose has demonstrated good performance in numerous applications, including paper making [13], water [14] and wastewater treatment [15], biomedical engineering [16], and energy production [17]. Different types of nanocellulose were first mentioned in articles by Ranby [18] and Turbak et al. [19], not only as a concept for scientific theoretical discussion, but also to report actual new cellulose products known as cellulose nanocrystal (CNC) and microfibrillated cellulose (MFC) or cellulose microfibril (CMF), respectively. Decades later, nanocellulose of various nanoparticle-size distribution and structure is still being produced by numerous active organizations until today.

As a case in point, the potential applications of nanocellulose are gaining a significant level of attention and have been extensively reviewed. Recent studies have significantly contributed to the understanding of the many properties, applications, and functionalization of nanocellulose. To date, most reviews are focused on only a specific type of nanocellulose-based material (either aerogel sorbents or adsorbents only) [11,20–21] or a specific type of treatment under environmental remediation (e.g., water treatment only) [22–23]. This review aims to provide an overview of the recent development and prospects of various nanocellulose-based materials used in various applications under the category of environmental remediation, including past achievements, current advances, and potential directions. The recent developments of nanocellulose used as an adsorbent, photocatalyst, flocculant or membrane for various applications in environmental remediation are detailed in this review.

### Categorization of nanocellulose

**Figure 1:**
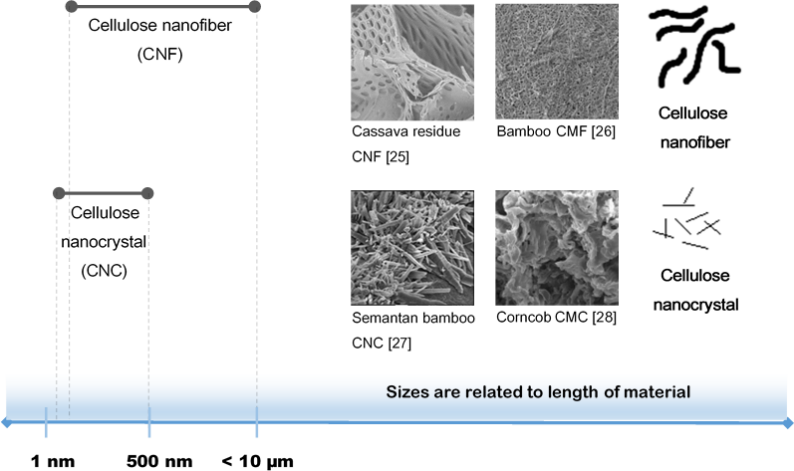
Size chart of nanocellulose based on material length (with microscopic images of various nanocellulose sources). Reprinted in part from Huang et al. [25], Nguyen et al. [26], Razalli et al. [27], and Kunusa et al. [28].

Cellulose nanomaterials (nanocellulose) can be categorized into two major groups based on its size and structure: nano-objects and nanostructures [24]. The two major groups can be branched down into specific subgroups: cellulose microcrystal (CMC) and cellulose microfiber under nano-object cellulose, and cellulose nanocrystal (CNC) along with cellulose nanofiber (CNF) under nanostructured cellulose. The main difference between nano-object cellulose (10–100 µm) and nanostructured cellulose (1–50 nm) is the size of the cellulose nanomaterial. Figure 1 illustrates the difference in length of CNF and CNC alongside with a few examples of microscopic images of nanocellulose derived from plants [25–28].

However, the Technical Association of the Pulp and Paper Industry (TAPPI) and multiple concerned bodies have recommended that nanocellulose be categorized into two main groups: CNC and CNF [20]. Overall, there are no fixed definitions for each group, since many nanocellulose sources coexist in the extensive and overlapping material space [29].

#### Cellulose nanofiber (CNF)

**Figure 2:**
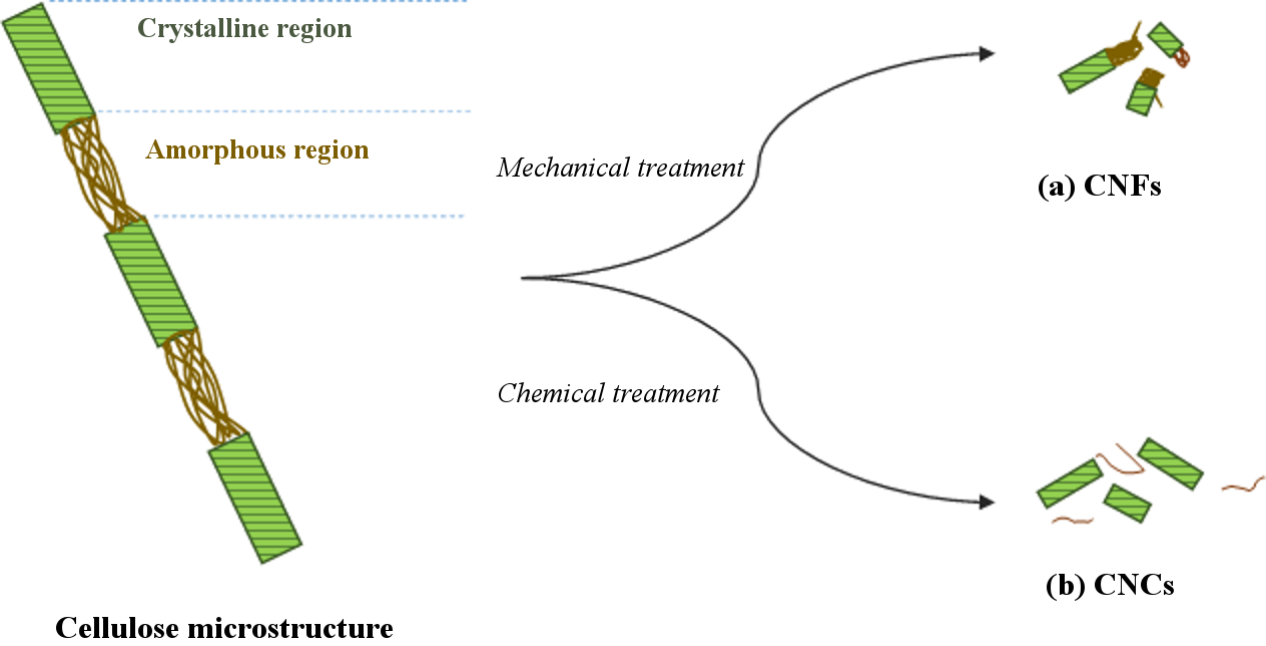
The structures of (a) cellulose nanofibers (CNFs) and (b) cellulose nanocrystals (CNCs) produced from cellulose.

Various terms have been used interchangeably with CNF, such as nanofibrillated cellulose (NFC), nanofibrillar cellulose, nanofibrous cellulose, and bacterial nanocellulose (BC) [24,29–30]. CNFs can be distinguished through their structure which is comprised of stretched masses of elementary nanofibrils with alternating crystalline and amorphous domains [24] (Figure 2a).

Due to the complex structure of cellulose fibre, the plant cell wall must be subjected to strong mechanical disintegration before turning the fibres into CNF [24]. The diameter of the fibres is commonly within the range of 10–100 nm, depending on the disintegration power [24]. In the context of CNF extraction from feedstocks, numerous mechanical techniques have been applied as discussed later. To our knowledge, BC is the only cellulose synthesized using the bottom-up approach via enzymatic synthesis. It is one of the purest forms of cellulose produced by non-photosynthetic organisms through enzymatic polymerization of organic substrates such as sugar and glycerol [11,31]. The main difference between BC and other plant-derived CNFs is the absence of certain functional groups (except alcohol) and polymers (lignin, hemicellulose, and pectin) in BC [32]. Therefore, BC is known for its high-purity in CNF form as well as high water-retaining capacity and crystallinity (80–90%) among the various types of CNFs [11].

#### Cellulose nanocrystal (CNC)

CNC goes by a few other terms: nanocrystalline cellulose (NCC) and cellulose nanowhisker (CNW). In contrast to CNF, CNC is less flexible due to its higher crystallinity (Figure 2b). This is because CNCs have an elongated rod-like shape made up of crystalline regions isolated from CNFs [24]. Its assembly occurs through a series of alterations, beginning with the excision of amorphous regions in CNFs to isolate the crystalline regions, which is categorized today as CNC [24]. Conventionally, this process is achieved using concentrated acid via acid digestion prior to additional treatment methods [33]. Chemical treatment methods are predominantly used as the main treatment method for CNC extraction. Overall, multiple post-treatments and purification steps must be taken to extract and obtain CNC.

### Sources of nanocellulose

Cellulose, generally speaking, has to be extracted from a source before nanocellulose can be produced. To date, cellulose can be obtained from a broad range of sources including algae, bacteria, plants, and tunicates. The source of the cellulose determines not only its size and properties, but also the energy consumption of the extraction process to produce nanocellulose [24]. Although it was reported that the source of cellulose only plays a minor role on the final properties of CNF [34], this is probably process-specific and true only under certain conditions [35]. Conversely, CNC properties are source-dependent, thus suitable cellulosic raw material and appropriate extraction techniques must be established based on the required final CNC properties and applications [33]. Furthermore, the environmental impact associated with the sourcing, extraction, processing, and use of raw materials used to fabricate products for environmental applications should be taken into consideration [36].

#### Algae

Much progress towards environmental sustainability has been made by utilizing the upcoming secret weapon of green energy: algae. Recent research on artificial photosynthesis such as CO_2_ fixation and water splitting has boosted the appeal of algae [37]. Significant attention has been given to the use of algae to fabricate adsorbents and membranes from its extracted nanocellulose for environmental remediation. The green alga, known by its scientific name *Cladophora*, is also known for its use for nanocellulose production. *Cladophora* green algae is also known as the *Shiogusa* seaweed in Japan. The use of these green filamentous algae for nanocellulose production is appealing as it can help resolve issues concerning water pollution in coastal areas. Nicolai and Preston’s findings offer compelling insights into the differences in cell-wall characteristics of various algae species, concluding that most *Cladophorales* algae are highly crystalline [38]. According to Mihranyan [39], the highly crystalline characteristic of *Cladophora* cellulose suggests higher inertness of its cellulose which reduces its susceptibility to chemical treatments compared to most native cellulose derived from conventional land plants. The filters fabricated from *Cladophora* algae cellulose have been tested and proven for trapping swine influenza virus particles with retention efficiency that matches industrial filters [40]. In addition, adsorbent beads have been fabricated using *Cladophora* cellulose for fast adsorption of palladium (II) ions of up to 80% maximum capacity in 2 h [41]. The initiation of this study could develop an alternative for electronic waste management by recovering and recycling palladium through an efficient and low-cost technology [42]. Algae as a supply for nanocellulose production could increase the production of cellulose significantly in an inexpensive manner and reduce greenhouse gases by absorbing carbon dioxide compared with bacteria [43].

#### Bacteria

Building on the multifunctional and environmental benefits of nanocellulose materials, many researchers have started to investigate the capacity for nanocellulose production through microbial hosts. Similar to algae-based cellulose, BC is of high purity as it is free from other polymers and functional groups except alcohol [32,44]. According to Gatenholm and Klemm [45], a typical BC production process can take up to two weeks to produce cellulose. Furthermore, nanocellulose produced from BC possesses higher crystallinity than plant-derived nanocellulose [46]. Over the decades, the types of sources used for cellulose production has varied widely. So far, cellulose produced by one of the few common bacterial species, such as *Acetobacter xylinum* (presently *Gluconacetobacter xylinus* or *Komagataeibacter medellinensis*) and *Gluconacetobacter medellinensis,* were investigated for their potential as adsorbent, catalyst, and membrane. BC-based adsorbent, catalyst, and aerogel membrane were applied for the removal of copper and lead [47], dye removal [44], and membrane distillation [48]. Ultimately, the current goal in BC production is linked to resolving the challenging aspect of its fermentation process which requires new cost-effective culture mediums to facilitate high BC production within short periods of time [49].

#### Plants

Plant cellulose fibres are one of the most extensively studied and used main sources for nanocellulose fabrication. Usually, it is chosen over BC and tunicate-based cellulose when thinner nanofibers are required for its applications [50]. According to García et al. [33], plant fibres used as cellulose sources can be classified into six groups: bast fibres, core fibres, grass and reed fibres, leaf fibres, seed fibres, and other fibres. The most common type of plant fibre used for cellulose fabrication is wood pulps (categorized under other fibres) due to its relatively high cellulose purity, durable and ductile networks, and good physical properties compared with other plant-based sources [51]. In the past decade, wood pulp nanocellulose has been used to fabricate catalysts for the reduction of 4-nitrophenol [52] and microfiltration membranes for bacteria, virus, and heavy metal ion removal [53]. In addition, bleached birch fibres from *Betula verrucosa* and *B. pendula* were also investigated in a study led by Suopajärvi et al. [54] to fabricate dicarboxylic acid nanocellulose flocculant and examine its effectiveness in the treatment of municipal wastewater (removal of turbidity and chemical oxygen demand (COD)). In another study, cotton microfibers were subjected to acid treatment, washing, and filtration to produce nanocellulose adsorbents for the removal of cadmium and nickel ion from water [55]. In view of these recent developments, there is much that plant-based nanocellulose can offer to green chemistry and biomaterial sciences for environmental remediation.

#### Tunicates

Nanocellulose can also be obtained from tunicates (sea or marine invertebrate animals). Many studies have identified the use of sea squirts in particular as a contemporary alternative for nanocellulose fabrication. In general, cellulose is formed on the outer tissue of the tunicates (*tunic*) and is comprised of highly pure and crystalline cellulose of CI_β_ allomorph type [24]. A number of sea squirts species, such as *Halocynthia roretzi* [56], *Halocynthia papillosa* [57], and *Metandroxarpa uedai* [58], have been used by researchers to produce CMF. Several attempts have been made to employ tunicate nanocellulose for environmental remediation. For example, Cheng et al. [59] has synthesized a highly efficient filter membrane for oil/water separation by combining super hydrophilic tunicate cellulose nanocrystal and cholesteric liquid crystal structure. The tunicate cellulose was derived from *Halocynthia roretzi drasche*, also known as the sea pineapple. In addition, nanocomposites fabricated from another species known as the stalked sea squirt (*Styela Clava*) has also demonstrated its usefulness as a catalyst for water remediation [60]. In a separate investigation led by Yu et al. [61], stalked sea squirt nanocellulose were also used as an environmentally friendly and inexpensive flocculant to flocculate and harvest microalgae for biodiesel production.

### Preparation techniques of nanocellulose

**Figure 3:**
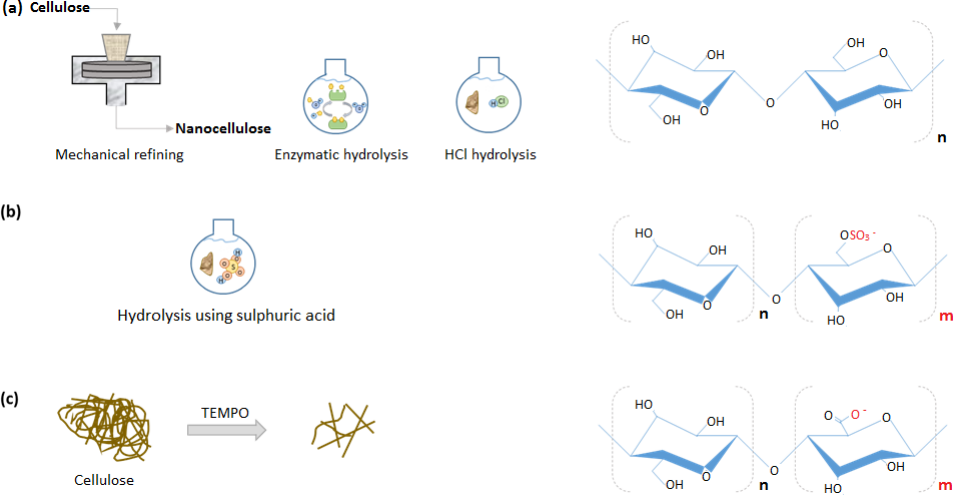
Resultant chemical structures of nanocellulose prepared via (a) mechanical refining, enzymatic hydrolysis, hydrolysis using hydrochloric acid, (b) hydrolysis using sulphuric acid, and (c) TEMPO-oxidation.

Prior to nanocellulose production, most cellulose is isolated from untreated cellulose pulp which contains hemicelluloses and lignin. Further processing on the purified cellulose pulp is required to produce CNF/CNC. In general, most CNCs and CNFs are produced through breaking down the cellulose fibres into nanosize fragments (top-down process), except for BC and electrospun cellulose nanofiber (ECNF) which utilize bacteria and an electrospinning technique (bottom-up process), respectively [62]. Typically, the production techniques employ several processes in sequence to produce various types of CNF/CNC which differ in terms of morphology, crystallinity, and surface chemistry [32]. These processes involve mechanical disintegration as well as biological and chemical pretreatments (Figure 3). The relevant techniques used to produce nanocellulose from purified cellulose are discussed briefly in this section.

#### Mechanical disintegration

In general, the majority of literature focuses on mechanical treatment as the principal treatment to produce CNFs [20] while it is also used as post-treatment and purification steps for CNC production [33]. Mechanical disintegration is commonly used to break cellulose pulp into smaller particles. However, efficient mechanical disintegration of cellulosic fibre normally requires elementary fibril delamination of cellulosic fibre instead of merely fibre shredding [32]. The occurrence of fibre shredding during mechanical disintegration of dry cellulose pulp tends to produce nanocellulose with poor mechanical properties. For improved delamination of nanofibrils, an aqueous medium is employed during mechanical disintegration to loosen interfibrillar hydrogen bonding and avoid reverse coalescence or fibril aggregation [32]. Several examples of commonly used techniques for efficient delamination of cellulosic fibre include homogenization [63], grinding [64–65], and refining [66]. Unlike finely structured or short rod-like nanocelluloses produced via chemical pretreatment, mechanically disintegrated nanocellulose is clustered (agglomerated) with larger dimensions [66].

According to Park et al. [67], the chemical composition in wood-based CNF has an effect on the defibrillation efficiency in wet disk-milling, which improved defibrillation in the absence of lignin and hemicellulose in cellulose. The transition from micro- to nanoscale during mechanical disintegration of fibres is usually evident in a reduction of viscosity, thermal stability, and crystallinity. In most cases, the principal mechanical treatments used are either aqueous counter collision, ball milling, blending, cryocrushing, electrospinning, extrusion, grinding, homogenization, refining, steam explosion, ultrasonication, or a combination thereof.

#### Chemical reaction

Effective and energy-efficient nanocellulose preparation techniques are being pursued to sustain and meet the high volumetric output of large-scale production. As a result, the chemical approach has emerged as a strong contender. According to Fan et al. [68], the usage of chemical agents is a significant contributor to the defibrillation process in the nanocellulose production.

Chemical treatments are used to isolate cellulose fibres to generate nanocellulose. Existing chemical pretreatment techniques include acid hydrolysis [69], carboxylation [70], carboxymethylation [71], quaternization [72], sulphonation [73], ionic liquid [74], and solvent-assisted pretreatments [75].

Acid hydrolysis is a common technique used to extract CNC from cellulose. Unlike mechanical disintegration, this technique destroys the amorphous region (non-crystalline region) in microfibrils, leaving the crystalline regions intact. Liu et al. [76] have demonstrated that CNC obtained through sulphuric acid hydrolysis (with –SO_3_^−^ functional group) (Figure 3b) showed superior Ag(I) adsorption capacity over CNC obtained via mechanical grinding. Similar to CNC extraction from cellulose, acid hydrolysis is commonly used to fabricate bacteria nanocrystals from BC microfibrils [77].

To aid in the delamination of nanofibrils in the subsequent step of mechanical disintegration, researchers have devised several chemical pretreatment techniques as supplemental steps to prepare nanocellulose. These techniques involve carboxylation, carboxymethylation, sulphonation, and quaternization to introduce surface charges onto the cellulose. Surface charge modification by carboxylation, carboxymethylation, and sulphonation introduces anionic functionality to aid the defibrillation of cellulose fibres based on the principle of electrostatic repulsion between similar charges [32]. Wågberg et al. [78] revealed that carboxymethylation of CNFs produced thin nanofibrils (3–15 nm) with minimal aggregation and improved uniform width distribution when compared to CNFs with no chemical pretreatment. Other methods like carboxylation via 2,2,6,6-tetramethylpiperidin-1-oxyl (TEMPO)-mediated oxidation (Figure 3c) have been shown to improve the delamination of nanofibrils [32] and produce well-defined individual CNFs when coupled with mechanical treatment. Recently, oxidative sulphonation of cellulose using periodate and bisulphate has been considered a potential green technique to promote nanofibrillation of cellulose compared with TEMPO-mediated oxidation. Sulphonation of cellulose provides lower anionic charges, broader functionalities, and consumes less harmful and expensive chemicals in its recyclable process which does not generate halogenated waste [79]. In contrast, quaternization has been used to provide cationic functionality to nanocellulose, which could be particularly useful for particle aggregation of negatively charged pollutants to coagulate and flocculate kaolin colloids as reported by Liimatainen et al. [79].

On the other hand, the utilization of green solvents (ionic liquids) as both solvent and catalyst for cellulose hydrolysis has become a popular alternative method for nanocellulose extraction. The extraction of nanocellulose using ionic liquids, such as 1-butyl-2-methyllimidazolium hydrogen sulphate (BmimHSO_4_), has been considered as a green approach for its economical process (due to high recovery of ionic liquid for reuse) and zero hazardous waste products [80].

#### Biological reaction

To combat challenges pertaining to high energy cost (linked to mechanical disintegration) and environmental concerns (linked to chemical pretreatment), enzymatic hydrolysis is introduced as an additional pretreatment step to reduce chemical waste and energy consumption. Enzymatic hydrolysis is commonly applied before mechanical disintegration of cellulose (refining or blending) in nanocellulose production. The addition of mono-component endoglucanase enzymes has proven to promote cell wall delamination during mechanical disintegration of cellulose in a homogenizer [81]. Furthermore, nanofibers produced via enzymatic treatment using endoglucanase were proven to yield better structure in terms of average molar mass and larger aspect ratio than nanofibers produced from acid hydrolysis (chemical treatment) [82].

Several other enzymes have been used for the preparation of nanocellulose: (1) cellulases [83], (2) pectinases [84], (3) xylanases [85], and (4) ligninases. Beltramino and co-workers [83] demonstrated that the application of cellulase as an enzyme substrate in the presence of acetate buffer was able to generate CNC with greater crystal dimensions and reduced sulphur content from cotton linter. Conversely, chemically treated banana fibres showed larger crystallinity (300%) in nanofibers than nanofibers treated with xylanase (200%) [85] since the enzyme showed difficulty in solubilizing the hemicelluloses and could not penetrate the cellulose chains to undergo efficient cellulose hydrolysis. Hemicellulose is a common component found in cellulose sources which hampers the separation of cellulose nanofibrils. Recently, Hu et al. [86] combined the use of endogluconase, lytic polysaccharide monoxygenase (LPMO), and xylanase as enzymes before ultrasound treatment for the nanofibrillation of cellulose derived from Kraft pulp. “Accessory enzymes” such as LPMO and xylanase were used to boost the hydrolytic performance of cellulose cocktails while endoglucanase facilitated the refining and fibrillation of cellulose. Overall, the potential of increasing substrate accessibility was made possible by the synergistic effect of the enzymes. On the other hand, Nechyporchuk et al. [32] reported that the rheological behaviour of CNF suspensions produced with enzymatic pretreatment showed superior flocculation ability compared with CNF produced from chemical pretreatment.

### Nanocellulose-based materials for environmental remediation

The global demand on utilizing sustainable and ecologically friendly products from natural resources such as cellulose has paved the way for intensive research on the potential application of micro- and nanometre-sized cellulose materials. Cellulose has been mainly used as reinforcement for plastic and paper industries while at the same time its application in the environmental field has recently aroused much attention. Nanometre-sized cellulose materials or nanocellulose has acquired an advantage over conventional cellulose fibres due to its higher surface area, aspect ratio, and Young’s modulus [87]. The versatility of cellulose-based materials has opened up doors for its use in a wide range of applications. In this section, an overview on the recent advanced applications of cellulose-based composites as adsorbents, photocatalysts, flocculants, and membranes in the field of environmental remediation will be reviewed.

#### Nanocellulose-based adsorbents

Wastewater purification using cellulose-based adsorbents materials has been developed as an alternative to the energy intensive and expensive technology based on commercial, activated carbon-based adsorption (Table 1).

**Table 1:** Cellulose-based biopolymer adsorbents for various types of organic pollutants.

Biopolymer adsorbent	Model pollutants	Adsorption capacity (mg/g)	Duration (min)	Ref.

calcium hydroxyapatite microfibrillated cellulose	Cr(VI)	114.79	5	[88]
magnetic Fe_3_O_4_@SiO_2_@cellulose composite	Cr(VI)	171.5	30	[89]
aminopropyltriethoxysilane-modified microfibrillated cellulose	Ni(II) Cu(II) Cd(II)	159.88 200.17 471.58	300	[90]
cotton fibre functionalized with triethylenetetramine and carboxymethyl chitosan	Pb(II) Cu(II)	144.93 95.24	120	[91]
poly(itaconic acid)–poly(methacrylic acid)-grafted-nanocellulose/nanobentonite composite	Co(II)	350.8	120	[92]
	U(VI)	121.02	120	[93]
MnO_2_-loaded biocomposite based on microcrystalline cellulose	Pb(II)	247.5	180	[94]
sulphonated cellulose	Fe(III) Pb(II) Cu(II)	98.7 83 47.5	30	[95]
sulphonated nanofibrillar cellulose	Pb(II)	248.6	1200	[96]
cationic cellulose nanofibers functionalized with glycidyltrimethylammonium chloride	PO_4_^3−^ SO_4_^2−^ F^−^ NO_3_^−^	55 50 10.6 44		[97]
nanocellulose-amino linked by maleic acid supported on magnetite	arsenic	85.3	90	[98]
Fe_3_O_4_@cellulose core–shell magnetic biopolymer	congo red	131	11	[99]
amino-functionalized nanocrystalline cellulose	acid red GR congo red 4BS reactive light yellow K-4G	134.7 199.5 183.0	300	[100]
carboxylate-functionalized adsorbent based on cellulose nanocrystals	crystal violet methylene blue malachite green basic fuchsin	243.90	240	[101]
functionalization of cellulose with hyperbranched polyethylenimine	congo red cationic basic yellow 28	2100 1860	60	[102]
cellulose nanocrystal–alginate hydrogel beads	methylene blue	255.5	210	[103]
cellulose aerogel based on cationic cellulose nanofibrils	blue dye CR19 red dye 180 orange dye 142	230 160 560	80	[104]

Unmodified cellulose usually does not exhibit good adsorption performance, therefore it needs to be specifically tailored to provide selective and enhanced interactions for a positive outcome. The essential and fundamental characteristics of cellulose include its hydrophilicity, functionalization ability, and ease of tunability of various properties like surface area, aspect ratio, quantum size effects as well as chemical accessibility [105]. Nanocellulose has been recognized as an excellent natural biomaterial adsorbent for wastewater treatment due to its adsorption affinity towards organic pollutants. Unlike micrometre-sized cellulose, the nanometre-sized counterparts are relatively smaller in dimensional size but also possess a larger surface area with improved porosity, which limits internal diffusion and quantum size effects [106]. The improvement in adsorption removal efficiency of nanocellulose could be due to the increased surface area, crystalline nature, and number of available functional moieties. Taleb et al. [98] reported that the introduction of amino-terminal functionalities on MFC and nanocellulose surfaces can improve their function as magnetite-based adsorbents for arsenic removal. It is proven that amino groups accessible for iron coordination result in a larger number of adsorption sites on the nanocellulose surface as compared to MFC.

In addition, nanocellulose has also been proposed as an adsorbent of residual antibiotics which are commonly found in industrial discharge, aquaculture, and effluent with medicinal residues. By converting cellulose to nanocellulose, the surface area increases exponentially along with its crystalline nature and the number of surface hydroxy groups (–OH) [101]. The abundant surface –OH groups act as hydrophilic sites to accelerate nucleation and growth of inorganic particles, consequently controlling the morphology, particle size, and crystallinity [107]. Such readily available surface –OH groups offer an ideal platform for the fabrication of hybrid nanocomposites with hierarchical structure. Besides, these –OH groups can be tailored for selective and improved interactions through a variety of chemical modifications [88]. The hydrophilicity of nanocellulose can be improved by inserting hydrophilic groups, such as carboxylic acids, alcohols, and amides, into its polymer chains. Reactive –OH groups are proven to enhance the adsorption behaviour of modified nanocellulose, especially towards organic contaminants. However, the hydrophilic nature of nanocellulose might cause it to aggregate during the drying process due to the greater number of –OH groups on the surface [93]. Therefore, an overabundance of –OH groups can produce an opposing effect, which is not ideal for wastewater treatment.

Among the hydrophilic functional groups, the functionalization of nanocellulose with carboxy groups through carboxylation can improve its adsorption performance for the removal of toxic metal ions from wastewater. A carboxy group has two lone pairs of electrons on the oxygen, and hence it is required to form a chelate with a divalent metal. In this regard, Qiao et al. [101] synthesized a carboxylate-functionalized adsorbent by grafting maleic anhydride on primary –OH of CNC. This modified nanocellulose adsorbent was used for the removal of cationic dyes from aqueous solution by forming a bidentate arrangement between the dye and the adsorbent’s carboxy groups. Meanwhile, Anirudhan et al. [92–93] modified a nanocellulose/nanobentonite composite adsorbent with multiple carboxy functional groups for the adsorption of uranium(VI) and cobalt(II). The co-polymerization of itaconic acid and methacrylic acid was able to introduce three carboxy functionalities which increased reactivity and hydrophilicity for effective removal of the metal ions from the aqueous solution. The adsorption capacity of the resultant adsorbent increased with increasing surface carboxylic group concentration of the adsorbent. A large amount of strongly ionisable COO^−^ groups could be generated through functionalization with poly(acrylic acid) [105]. Besides, carboxylated nanocellulose displayed high uptake adsorption capacity for several cationic dyes such as methylene blue, basic fuchsin, crystal violet and malachite green [101]. Carboxylated CNC, with a high COOH content of 2.1 mmol/g, showed a significant uptake of about 769 mg/g for methylene blue as compared to CNC with surface sulphate groups.

Alternatively, amine modification aids the chelating action on the desired pollutant anions, thereby enhancing the adsorption capacity of the nanocellulose adsorbent materials. Metal ions can adsorb onto the adsorbent via electrostatic interaction and ion exchange since amino groups are easily protonated under acidic conditions [89]. The amino group possesses a lone pair of electrons which is attributed to the nitrogen atoms and may form a covalent bond with metal ions. The adsorption of metals ions is coordinated by amino and hydroxy groups found in the functionalized cellulose. Amine functional groups usually induce a high adsorption rate at low pH while a decrease in the adsorption uptake capacity is observed in medium with pH above 8. The aminopropyltriethoxysilane modified MFC’s affinity for different metal ions follows the order of Cd(II) > Cu(II) > Ni(II) [90]. The modified nanocellulose materials were able to adsorb metal ions due to the presence of amino (–NH_2_) on aminosilane and/or –OH groups on cellulose fibre. Besides, cationic functionality such as amine-functionalized nanocellulose is usually effective for the removal of anionic dyes. Zhu et al. [102] found that functionalization of cellulose with cationic hyperbranched polyethylenimine presented high adsorption capacity for congo red (2100 mg/g) and basic yellow (1860 mg/g).

Sulphonated cellulose possesses a high amount of sulphur binding sites, high anionic charge, and larger surface area, thereby displaying a high affinity towards multiple metal ions and an increase in adsorption [95]. Dong et al. [95] discovered that the order of removal and regeneration efficiencies of sulphonated cellulose for three metal ions were in the order of Fe(III) > Pb(II) > Cu(II) and Cu(II) > Pb(II) > Fe(III), respectively, and hence consistent with its affinity to the adsorbent. It has been reported that the sulphonic group has a larger degree of ionization than other functional groups such as hydroxy and carboxy groups, and thus the electrostatic affinity of the sulphonic group to metal ions is stronger [96,108].

In addition, Sehaqui et al. [97] found that the adsorption capacity of cationic CNFs toward anions increased with the surface charge content of cellulose. They concluded that cationic CNFs with an ammonium content of 1.2 mmol/g can be considered as an efficient adsorbent for negatively charged ions such as nitrate (NO_3_^−^), phosphate (PO_4_^3−^), fluoride (F^−^), and sulphate (SO_4_^2−^). Cationic CNFs exhibited higher selectivity towards multivalent ions (PO_4_^3−^ and SO_4_^2−^) than monovalent ions (F^−^ and NO_3_^−^).

On the other hand, the development of nanocellulose composites as adsorbents with numerous supportive properties, magnetic nanocellulose for example, is desired due to their good performance in the removal of organic pollutants. Hokkanen et al. [88] reported that calcium hydroxyapatite-microfibrillated cellulose adsorbent was effective for the removal of chromium (Cr(VI)) from aqueous solution. They found that calcium hydroxyapatite nanostructures can interact with cellulose structures effectively, resulting in the enhancement of nanocomposites properties due to their strong hydrogen bond, divalent character, and relatively small ionic radius. Anirudhan et al. [92] chemically modified a composite polymer to produce a poly(itaconic acid)–poly(methacrylic acid)-grafted-nanocellulose/nanobentonite composite as an efficient adsorbent for the removal of cobalt (Co(II)) from aqueous solution. This could increase the availability of the multi-carboxy functional moiety, which is covalently bonded to the inorganic matrix, to effectively bind metal ions.

Conventional cellulose-based adsorbents are difficult to recover from treated wastewater. The recovery step usually requires filtration or high-speed centrifugation. In contrast, magnetic cellulose nanocomposites can easily overcome the recovery issue by application of an external magnetic field. As a result, magnetic nanomaterials have drawn increasing attention. Nanocellulose incorporated with other magnetic nanomaterials is presented as an excellent composite adsorbent with magnetic properties. For example, a core–shell cellulose magnetite (Fe_3_O_4_) polymeric ionic liquid magnetic biosorbent has been developed and employed for congo red adsorption [99]. The benzyl groups within the cellulose structures are responsible for the magnetic nature of the polymeric ionic liquid which was created via reaction with epichlorohydrin and 1-methylimidazole. In addition, the functionalization could increase its aromatic character, anion exchange capability, and hydrophilic nature of cellulose. Cellulose could also act as a stabilizer for the Fe_3_O_4_ nanoparticles to prevent crystal growth and aggregation of particles.

In general, magnetic iron oxide is anchored in the polymer matrix to induce magnetic properties in nanocomposites such as Fe_3_O_4_@SiO_2_@cellulose nanocomposite and amino-functionalized magnetic cellulose nanocomposite grafted with glycidyl methacrylate followed by reaction with ethylenediamine [89]. Furthermore, a 7 wt % NaOH/12 wt % urea aqueous solvent was found useful for regenerating Cr(VI) from the amino-functionalized magnetic cellulose nanocomposite. In short, the development of new biomaterials by combining two or more natural materials (or through functionalization of nanocellulose) have become increasingly interesting techniques to construct materials with combined properties which could not be achieved by the individual material alone.

The prospects for nanocellulose-based adsorbents in wastewater treatment have never looked better. But given the mixed results from studies (Table 1), and the practical challenges for implementation (treatment duration, upscaling, and material toxicology) for some cases, further optimization and improvement must be made. Furthermore, the fabrication of nanocellulose-based adsorbents is mostly established based on CNF, and limited when it comes to CNC.

#### Nanocellulose-based photocatalysts

Recently, numerous inorganic-organic hybrid-based nanostructured composites have emerged as new types of photocatalysts with remarkable optical, electrical, mechanical, and thermal properties. The gravitation from petrochemical-based feedstock to environmentally friendly biomaterials such as cellulose-based materials is making headway for a cleaner and more sustainable environment [109]. Cellulose-derived materials have been identified as an almost limitless source of chemical raw material as it is the most abundant renewable organic resource on earth. Cellulose has shown attractive potential as an environmental friendly and biocompatible support due to its exceptional properties of chirality, hydrophilicity, and broad chemical variability [110]. Besides, cellulose is usually considered as a good host material for nanoparticles because cellulose can improve the stability and control the growth of nanoparticles while retaining its special morphology [111].

Although cellulose alone exhibits limited photocatalytic activity under UV or visible light irradiation, many semiconductor materials have been added to enhance the photocatalytic activity. Several research groups have demonstrated photocatalytic wastewater treatment using cellulose-based metal oxide nanostructures in the form of thin film, membrane, fibre and hybrid materials under UV and visible light irradiation. Nanocellulose–metal oxide (TiO_2_, ZnO, graphene oxide, and Fe_2_O_3_) composites have been used as photocatalysts to improve the degradation rate of organic pollutants as compared to individual materials. Table 2 summarizes the application of cellulose-based photocatalyst nanostructured materials for photocatalytic degradation of various organic pollutants.

**Table 2:** Application of nanostructured cellulose-based hybrid photocatalysts in photocatalytic degradation of various types of organic pollutants.

Type of catalyst	Model pollutants	Light source	Photocatalytic degradation efficiency (%) and reaction time (min)	Ref.

graphene oxide and TiO_2_ nanoparticle incorporated alginate/carboxymethyl cellulose nanocomposite	30 mL of 30 mg/L congo red	solar light with average intensity of ≈900 W/m^2^	98%, 240 min	[112]
nanoscale zinc oxide incorporated graphene oxide/nanocellulose composite	25 mL 2–4 mg/L ciprofloxacin	solar light 100 mW/cm^2^	98%, 40 min	[113]
ZnO/cellulose nanocomposite	50 mL 3.25 g/L methylene blue	UV lamp with unknown wavelength and intensity	79%, 300 min	[114]
hydroxypropyl cellulose/molybdenum disulphide nanocomposite hydrogels	250 mL methylene blue	sunlight	90%, 180 min	[115]
TiO_2_–hydroxypropyl methyl cellulose nanocomposite	100 mL 10 ppm 4-nitrophenol	250 W halogen lamp (main range 400–800 nm)	85%, 180 min	[116]
graphene oxide/TiO_2_/bacterial cellulose nanocomposite	100 mL 10 mg/L methyl orange	UV lamp (365 nm, 175 W)	100%, 120 min	[117]
alginate/carboxymethyl cellulose/TiO_2_ nanocomposite hydrogel	30 mL 30 mg/L congo red	sunlight	91.5%, 240 min	[118]
nanocrystalline TiO_2_ on cellulose fabric	99.995% CO_2_ at flow rate 300 mL/min for 1 h	four UVA lamps (8 W each lamp) λ = 365 nm	194.0 ppm/g and 50.8 ppm/g for CO and CH_4_, 360 min	[119]
CdS nanoparticle/bacterial cellulose nanofibers	200 mL 20 ppm methyl orange	300 W Xe lamp with a cut-off of λ < 420 nm	82%, 90 min	[109]
cellulose fibre supported zinc phthalocyanine	10 mL of 50 μM basic green 1	100 W lamp visible light, λ > 400 nm	98%, 90 min	[110]
immobilized Cu_2_O nanoparticles in cellulose/graphene oxide composite film	10 mg/L methyl orange	sunlight (cloudy, 20 °C)	72%, 300 min	[120]
N-doped TiO_2_ nanorods in regenerated cellulose thin films	150 mL 40 mg/L methylene blue	UV lamp at λ = 312 nm, 30 W	96%, 360 min	[121]
electrospun cellulose acetate membrane supported Ag@AgCl	methyl orange	500 W Xe arc lamp equipped with a UV cut-off filter (λ > 420 nm)	73% , 160 min	[122]
immobilized TiO_2_ nanoparticles and laccase on bacterial cellulose membrane	3 mL 15 mg/L reactive red X-3B	UV irradiation with unknown wavelength and intensity	95%, 180 min	[123]
graphene oxide/TiO_2_ based ultrafiltration cellulose membranes	3.40 × 10^−5^ mol/L diphenhydramine at flow rate of ≈0.25 mL/min	UV irradiation 350 nm and 33 mW/cm^2^	65%, 240 min	[124]

Cellulose derivatives can easily adsorb on metal oxide surface layers and provide additional –OH groups on the surface of metal alkoxides [116]. As a result, the surface area of this hybrid structure is expected to increase and expand its wavelength response up to the visible range. Bacterial cellulose shows the ability to act as a metal oxide nanoparticle support due to its high surface-area-to-volume ratio with a fibre diameter in the range of 40–70 nm, high mechanical strength and super-hydrophilicity properties [123]. Its ultrafine three-dimensional network provides bacterial cellulose with a high specific surface area, owing to the well-separated nano- and microfibrils. Besides, the presence of –OH binding sites and a fibrous network can facilitate the adsorption of metal oxide to its surface. Thus, Li et al. [123] proposed the immobilization of TiO_2_ nanoparticles on a fine fibrous network of bacterial cellulose via hydrogen bonds and electrostatic adsorption effect for the photocatalytic degradation of reactive X-3B. TiO_2_ nanoparticles and cellulose chains are compatible due to covalent bonds, which can improve the rigidity of the polymer chain and increase the energy required to break down the polymer chain [121]. Similar enhancement results were also obtained with cadmium sulphide (CdS) nanocrystals deposited on NCC which was derived from microbial cellulose to act as a highly efficient photocatalyst [109]. The composite catalyst demonstrated a high photocatalytic degradation efficiency of about 82% after 90 min of irradiation and exhibited high recyclability of up to five catalytic cycles.

In addition, the potential of fibrillated cellulose as a support/carrier for catalyst nanoparticles has attracted a lot of attention in recent years. For example, a study led by An et al. [125] discusses the application of CNFs as a support for titanium dioxide nanoparticles to prepare nano-fibrillated cellulose–TiO_2_ nanoparticle nanocomposites for photocatalytic hydrogen generation. The as-prepared nanocomposites generated 2–5 more hydrogen molecules in comparison with the control TiO_2_ nanoparticles. However, they found that the nanocomposites tend to photodegrade during photocatalytic reaction, and hence some of the nanocomposite particles will be lost during the recycling process. In another study, Taranto et al. [126] found that cellulose was sensitive to UV irradiation and prone to degradation during photocatalysis. The cellulose might compete with organic pollutants such as methanol for photocatalytically generated active species/sites, leading to a decrease in methanol reaction rate. Besides, Puls et al. [127] clarified that TiO_2_ could cause pitting on the surface of cellulose acetate/cellulose pulp fibres under UV illumination. Therefore, cellulose nanomaterials need to be coated and covered perfectly by inert UV-absorbing materials in order to protect cellulosic fibres from UV bleaching [128].

An et al. [129] claimed that the prepared nano-fibrillated cellulose/magnetite/titanium dioxide nanocomposites had a higher photocatalytic hydrogen generation rate as compared to the nano-fibrillated cellulose/titanium dioxide sample. The incorporation of magnetite was able to inhibit the photodegradation of the cellulose structure during UV irradiation and this hybrid structure demonstrated high catalyst recyclability using an external magnetic field. Nanoparticles generally tend to aggregate, leading to difficulties in recycling and reuse. However, a rigid cellulose matrix has been recruited for its three-dimensional porous structure to act as a catalyst support and prevent the aggregation and growth of nanoparticles [120]. The application of a nanocellulose-based thin film and membrane showed great potential in wastewater treatment due to its ease of recollection and recycling purpose, resulting in no photocatalyst residue in the reaction system.

Moreover, metal oxide nanoparticles of smaller particle size and larger surface area can be expediently immobilized in the cellulose film to obtain a metal oxide nanoparticles/cellulose nanocomposite. Photogenerated electrons and holes inside the metal oxide nanoparticles are able to migrate to the particle surface, while metal oxide nanoparticles within the nanocomposite film are also inclined to accelerate the interfacial charge carrier transfer and separation [130]. Mohamed et al. [121] reported that the optical properties of a transparent nanocellulose/TiO_2_ hybrid thin film were imperative for the UV or visible light irradiation. This led to the enhancement of the electron distribution and transfer to the surface of metal oxide. Consequently, the recombination rate of the photogenerated carriers decreased during the photochemical reaction.

It was reported that electrospun cellulose acetate nanofibrous membranes with large specific surface area, high porosity, and high permeability could be an effective support for photocatalysts [122]. This could create a synergistic effect that enhanced the catalytic performance of the individual materials. Besides, Pastrana-Martínez et al. [124] observed that three photocatalysts, i.e., commercial TiO_2_ P25, lab-made TiO_2_, and graphene oxide doped TiO_2_ (GO-TiO_2_), which were assembled on flat sheet cellulose membranes, exhibited multifunctionality of water filtration and photodegradation of diphenhydramine and methyl orange. This study demonstrated the advantage of using a cellulose-derived film to support the TiO_2_ nanoparticle–cellulose film as a good support to avoid TiO_2_ loss during photocatalytic treatment of wastewater.

#### Nanocellulose-based flocculant

Common chemical conditioning is usually performed by using inorganic, multivalent cationic coagulants such as aluminium, iron salts or synthetic polyelectrolytes such as synthetic polyacrylamides. These synthetic polyelectrolyte materials pose environmental problems due to their derivatives, monomers or intermediate products that are hazardous, not readily biodegradable, and hold potential toxicity, which can cause secondary pollution to the environment [131]. Consequently, there is a need to look for suitable flocculants which are sustainable and natural bio-based alternatives for wastewater purification. These flocculants are expected to be degradable and prevent secondary pollution to the natural environment.

Biopolymer-based flocculants such as chitosan, tannins, cellulose and alginate are attracting wide interest from many researchers. Bio-based flocculants are not only known for being biodegradable but also have a high specific surface area and have dimensions in the nanoscale regime [132]. Commonly, there are two categories of natural polymer based flocculants [133]. One of these categories involves the grafting of natural polymers to fabricate semi-natural flocculants such as polyacrylamide-grafted hydroxypropyl methyl cellulose [134]. The other type is derived by directly modifying natural polymers to construct improved natural flocculants such as dicarboxylic acid nanocellulose [54].

Reports on the usage of nanocellulose as water chemicals are still rare since researchers mainly focused on adsorption of metals from diluted aqueous solution or photocatalysis as discussed in previous sections. Agglomeration remains an issue in the application of CNCs as flocculants. This is due to the hydrogen bond networks arising from the –OH groups on the CNCs’ surface [135]. However, the properties of CNC flocculants can vary with changes in the surface –OH group, and hence the right amount of –OH surface groups must be established to produce an effective flocculant. Anionic surface groups such as hydroxy groups are commonly found in nanocellulose after pulp processing and due to the presence of compounds from the plant cell wall [79]. Consequently, the interaction and compatibility among anionic nanocellulose and several inorganic minerals are commonly negatively charged and weak. It has been reported that an anionic flocculation agent possesses poor solubility in acidic solution and hence, limits its practical application as a flocculant in these conditions [136].

Therefore, chemical pretreatments such as carboxymethylation [79], periodate-chlorite oxidation [79,131], aminoguanidine-reacted wood celluloses [131], 2,2,6,6-tetramethylpiperidin-1-oxyl-mediated (or TEMPO-mediated) [137], and citric/hydrochloric acid hydrolysis [135] are used to increase the anionic charge density or provide cationic charges to the nanocellulose, which has diversified the application of nanocellulose. For example, the modification of nanocellulose to produce such cationic counterparts are especially useful for the removal of anionic particles through molecular interaction of opposing charges which promotes particle aggregation in wastewater treatment.

Currently, functionalization of nanocellulose to generate effective flocculants is carried out by introducing anionic, cationic, or hydrophobic functional groups to the nanocellulose surfaces through a charge neutralization mechanism. The –OH groups on the cellulose surface enables facile functionalization of nanocellulose to introduce the desired functionality and produce highly effective flocculants [138]. The interaction of cellulose with various compounds could be enhanced after introducing new functional groups in order to increase the surface polarity and hydrophilicity [87]. Reported applications of cellulose-based flocculants in wastewater treatment are summarized in Table 3.

**Table 3:** Application of cellulose-based flocculants in wastewater treatment.

Flocculant	Model pollutant	Analytical test	Optimum result (%)	Ref.

anionic sodium carboxymethylcellulose	natural surface water	turbidity	93	[70]
anionic dicarboxylic acid nanocellulose	municipal wastewater	turbidity COD	80 60	[58]
crystalline nanocellulose grafted with cationic pyridinium functional groups	freshwater microalgae	microalgae biomass	95	[71]
nanofibrillated into cationic nanocelluloses	activated sludge	turbidity COD	90 60	[53]
cationic dialdehyde cellulosic nanofibrils	kaolin wastewater	colloid aggregation	95	[60]
anionic sulphonated nanocelluloses	municipal wastewater	turbidity COD	80 60	[87]
hydroxypropyl methyl cellulose grafted with polyacrylamide	mine wastewater	turbidity	94	[57]
cationic pyridinium cellulose nanocrystals	microalgal biomass	flocculation efficiency	100	[63]
cationic cellulose nanofibrils	municipal activated sludge	turbidity COD	90 20–70	[53]
anionic carboxylated cellulose nanocrystals	kaolin suspension	turbidity	80.9	[59]
rod-shaped cellulose nanocrystals	flocculation and phase separation of bacteria	aggregation percentage	100	[69]
poly(*N*,*N*-dimethylacrylamide) and polyacrylamide grafted cellulose nanocrystals	kaolin suspension	turbidity	69–91	[72]

Suopajärvi and co-workers had investigated various functionalized nanocellulose materials such as anionic and cationic dialdehyde as water chemicals in flocculation of real wastewater [54,87,131]. The results are promising and proved that functionalized nanocellulose could be employed as potential wastewater treatment agents. The modification on the surface groups on nanocellulose can be done by introducing anionic groups such (such as carboxy) to improve its effectiveness as an adsorbent by loosening the network form through surface hydrogen bonding [139]. Periodate oxidation has been applied for such modification to introduce aldehyde groups through oxidation of surface –OH groups and alteration of the carbon network of the glucopyranose ring [140]. These aldehyde groups of 2,3-dialdehyde cellulose can easily be converted further into various functional groups such as carboxylic acids, sulphonates or imines [87]. Liimatainen et al. [141–142] investigated the usage of anionic dialdehyde cellulose and cationic dialdehyde cellulose, derived via aqueous periodate oxidation. The study showed that anionic cellulose nanoparticles resulted in better flocculation performance of kaolin than that of cationic dialdehyde cellulose derivatives.

Another effective method to chemically modify nanocellulose to produce high charge material is by using TEMPO-mediated oxidation under aqueous and mild conditions. This method is suitable for the conversion of surface hydroxymethyl groups to their respective carboxylic forms [137]. The negatively charged groups on the surface of nanocellulose could prevent aggregation of nanocellulose via electrostatic repulsion. Chen et al. [143] reported that this electrosterically stabilized NCC can be produced by a three-step reaction: periodate, chlorite, and TEMPO oxidation. They claimed that periodate oxidation resulted in cellulose degradation. However, the cellulose degradation could be minimized by using less periodate oxidation and more TEMPO-mediated oxidation.

Many studies have focused on the modification of the physical and chemical structure to improve nanocellulose properties. For instance, Sun et al. [144] reported the effectiveness of applying CNCs to flocculate Gram negative bacteria (*Pseudomonas aeruginosa* PAO1). The study found that the flocculation efficiency based on the depletion of bacteria depended on the shape of the cellulose colloidal particles. In this case, rod-shaped particles (commonly found in CNC) could deplete larger colloids than spherical particles [145]. Besides, the introduction of cationic pyridinium and imidazole functional groups through grafting provided an advantage to CNCs over common polymer flocculants in terms of flocculation efficiency (flocculation of negatively charged microalgal cells) [138–139]. In short, researchers have been working to investigate various modifications of nanocellulose in order to broaden its industrial applications.

#### Nanocellulose-based membranes

In this section, the role of nanocellulose as a fabrication material in the field of membrane technology for environmental remediation will be discussed. Cellulose nanofibers and nanocrystals have been studied as membrane construction materials for wastewater treatment [146], gas separation [147], removal of dyes [148], removal of heavy metals through ion exchange [149], ultrafiltration [14,150], and adsorption [151]. The key information related to nanocellulose used in membrane technology for application in environmental remediation is summarized in Table 4.

**Table 4:** Application of nanocellulose material in membrane technology for environmental remediation.

Membrane application	Cellulose type	Membrane fabrication feature	Membrane type	Operating condition	Membrane performance	Ref.

wastewater treatment (to remove urea)	nanocellulose	metalized nanocellulose composites–thin film composite membrane (MNC-TFC)	forward osmosis membrane	crossflow forward osmosis	water flux (LMH/bar): 1. urea: 7.4; wastewater: 11.8 2. urea: 5.4; wastewater: 11.5 (both membranes performed better than a commercial HTI-CTA membrane)	[146]
removal of heavy metals (ion exchange of Cd(II) ions)	cellulose nanofibers	regenerated cellulose nanofiber membrane with poly(glycidyl methacrylate) (PGMA) coating and poly(acrylic acid) (PAA) grafting using ethanol or/and water as grafting solvent	ion exchange membrane	dead-end filtration	maximum Cd binding capacity, *Q*_max_ (mg/g): 1. 40.0 2. 39.8 3. 162.5 4. 138.7	[149]
carbon dioxide capture	cellulose nanofibers	casting of pure microfibrillated cellulose membrane (MFC) and MFC–polyvinylamin (Lup) 50/50 wt/wt nanocompositemembrane	gas permeation membrane	gas permeation with constant humidity control; temperature, 35 °C; low pressure condition, 1 bar	selectivity: 1. CO_2_/N_2_ 500; CO_2_/CH_4_ 350 2. CO_2_/N_2_ 35; CO_2_/CH_4_ 20	[147]
removal of metal ions (removal of Ag^+^, Cu^2+^ and Fe^2+^/ Fe^3+^ ions)	cellulose nanofibers; cellulose nanocrystals (cellulose sludge based (SL) and bioethanol based (BE)); phosphoryl group modified cellulose nanocrystal (PCNC), cellulose sludge based (SL)	support layer fabricated by vacuum filtration of cellulose nanofibers (CNF) and active layer fabricated by dip coating of cellulose nanocrystals (CNC) support layer fabricated by vacuum filtration of cellulose sludge (S-G) and active layer fabricated by further vacuum filtration of cellulose nanocrystals (CNC)	ultrafiltration membrane	crossflow filtration	removal (%): 1. 77% Ag^+^, 94% Cu^2+^ and 95% Fe^2+^/Fe^3^ 2. 91% Ag^+^, 99% Cu^2+^ and 100% Fe^2+^/Fe^3^ 3. 94% Ag^+^, 99% Cu^2+^ and 100% Fe^2+^/Fe^3^ 4. 100% Ag^+^, 13% Cu^2+^ and 14% Fe^2+^/Fe^3^ 5. 100% Ag^+^, 36% Cu^2+^ and 33% Fe^2+^/Fe^3^ 6. 100% Ag^+^, 86% Cu^2+^ and 74% Fe^2+^/Fe^3^	[14,150]
adsorption of metal ions	cellulose nanofibers; cellulose nanocrystals (cellulose sludge based (SL) and bioethanol based (BE)); TEMPO functionalized nanocrystals (bioethanol based (BE))	support layer fabricated by vacuum filtration of cellulose sludge (S) or cellulose sludge with nanocellulose nanocrystal (SL) and active layer fabricated by further vacuum filtration of cellulose nanocrystals (CNC); in situ TEMPO functionalization on active layer	ultrafiltration membrane	crossflow filtration	adsorption capacity (mg/g): 1. 0.81 Ag^+^, 250 Cu^2+^ and 384 Fe^2+^/Fe^3^ 2. 0.83 Ag^+^, 254 Cu^2+^ and 396 Fe^2+^/Fe^3^ 3. 0.86 Ag^+^, 339 Cu^2+^ and 416 Fe^2+^/Fe^3^ 4. 0.87 Ag^+^, 374 Cu^2+^ and 456 Fe^2+^/Fe^3^	[151]
removal of dyes	cellulose nanocrystals	casting of cellulose nanocrystals and chitosan solution	ultrafiltration membrane	dead-end filtration	removal (%): 1. 69–98% 2. 71–98%	[148]

**Figure 4:**
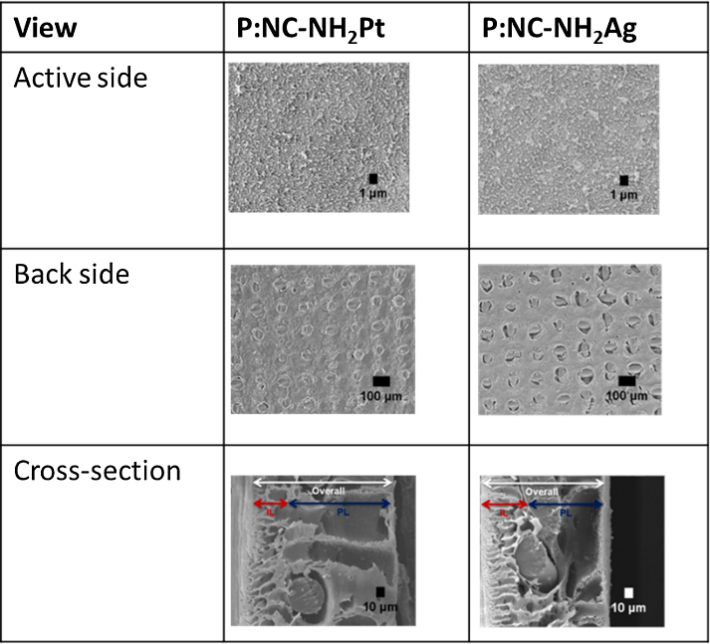
The morphology of MNC forward osmosis membranes with amino silane functionality as well as Pt and Ag nanoparticles. The ridge-and-valley morphology is commonly attributed to polyamide membranes. Reprinted (adapted) with permission from [146], copyright 2017 American Chemical Society.

Metalized nanocellulose composites (MNCs) used to construct the support of a forward osmosis membrane for wastewater treatment has been reported by Cruz-Tato et al. [146]. The nanocellulose was modified to incorporate amino silane functionalities before deposition of silver and platinum nanoparticles (Figure 4).

The fabricated MNC forward osmosis membrane performed better in terms of water flux when compared with a commercial HTI-CTA membrane. The total organic carbon rejection of the fabricated MNC membrane (94.6%) was found to be slightly lower than HTI-CTA (96.6%). On the other hand, Chitpong and Husson have prepared a high productivity, high capacity cation-exchange membrane for the removal of trace cadmium from water [149]. The performance of an ion exchange membrane in terms of cadmium binding capacity was found to be greatly influenced by the selection of grafting solvent. In other studies, Ansaloni and co-workers have taken the advantage of a unique feature of nanocellulose under humid conditions for carbon dioxide gas separation [147]. The gas separation membrane that was fabricated using pure MFC was found to be promising in terms of carbon dioxide selectivity (CO_2_/N_2_: 500; CO_2_/CH_4_: 350), especially when the controlled relative humidity is greater than 80%. The addition of polyvinylamin to pure MFC in the membrane construction improved the gas permeability consistency at a cost of selectivity (CO_2_/N_2_: 35; CO_2_/CH_4_: 20). According to Karim et al. [14], cellulose nanofibers can be used to fabricate a membrane through vacuum filtration with and without active layers of the membrane dip. The active layer membrane was prepared by coating both sides of the membrane surface with cellulose nanocrystals to form an ultrafiltration membrane for the removal of metal ions (Ag^+^, Cu^2+^ and Fe^2+^/Fe^3+^). In this case, acetone treatment was effective in controlling the pore structure, mechanical properties, and permeability of the nanocellulose membrane. High metal ion rejection and flux were achieved using the membrane that underwent acetone treatment. A subsequent study led by Karim et al. demonstrated the fabrication of membranes using vacuum filtration of cellulose sludge followed by cellulose nanocrystals [150]. The change in the membrane fabrication method resulted in improved removal of Ag^+^ with the with the consequence of reduced rejection performance of Fe^2+^/Fe^3+^. In situ TEMPO functionalization of the active layer of the nanocellulose membrane for the adsorption of metals ion was also reported by Karim et al. [151] as an innovative route to increase the membrane functionality without altering the bulk network structure. The metal ion adsorption ability, wettability, and negative surface zeta potential of the membrane was further enhanced after in situ TEMPO functionalization. In addition, the removal of positively charged dyes was reported using cellulose nanocrystals and chitosan through cross-linked and non-cross-linked treatment [148]. The mechanical stability, dimensional stability, and removal performance of the membrane has been further enhanced after the crosslinking process treatment.

As a construction material in the field of membrane technology for environmental remediation, nanocellulose is still at the development stage. To date, very limited literature is available and hence further study in this area is needed. This promising material could be used to replace or reduce the amount of non-renewable polymeric materials for membrane fabrication in the near future.

### Challenges and limitations

Although nanocellulose appears as a promising material for environmental remediation, there are still many challenges and drawbacks affecting the application of this nanomaterial. To promote the commercialization and marketability of nanocellulose for environmental remediation, the application must be easily scalable, low cost, and produce valuable end-products [20]. So far, most investigations on nanocellulose production are only on a laboratory scale. The challenges lies in the scalability, environmental impact, and cost that come with the selection of treatment routes to produce nanocellulose. Several commonly used techniques related to mechanical disintegration are suitable for upscaling. However, these techniques are inclined toward high energy consumption. Although other mechanical techniques are being studied to overcome this issue, most are still in its infancy and considered not suitable for upscaling. As an alternative, biological and chemical pretreatment techniques were developed to tackle this issue. On the contrary, chemical pretreatment does not necessarily reduce the energy demand for nanocellulose production according to a life cycle study done by Arvidsson et al. [152]. The study showed that the carboxymethylation process (chemical pretreatment) exhibited a higher cumulative energy demand than the no pretreatment route (homogenization only) due to the high input of chemicals such as ethanol, isopropanol, and methanol for CNF production [152]. In this case, chemical pretreatment of nanocellulose could actually result in a higher environmental impact due to the presence of solvent waste and acidic waste although lower electricity usage is required [20]. Another challenge would be the questionable large-scale production of BC and ECNF, which is difficult due to the nature of their production (low yield). This is because BC and ECNF production depends on the build-up of nanofibers from low molecular weight sugars by bacteria and dissolved cellulose by electrospinning, respectively [32]. The production strategies of nanocellulose still require continuous examination to construct environmentally responsible and economically feasible nanocellulose.

Furthermore, the economic and regulatory hindrances due to the unclear toxicology of various modified nanocellulose materials for environmental remediation may be the greatest obstacle for its application and marketability. The environmental and biological toxicity of cellulose nanomaterial is of utmost importance when one considers its use in environmental remediation. Eco-toxicology studies on nanocellulose-based composites are still limited and are at a primary stage [153]. In general, the danger of nanoparticles increases with decreasing particle size when inhaled as the small size eases uptake into cells, blood, and lymph circulation to reach potentially sensitive target areas [154]. Although most nanocellulose-based materials are non- (or only slightly) toxic, there have been reports of negative effects on living cell viability and proliferation [155], especially at high concentrations. In addition, it was recently reported that CNC could induce cytotoxic and inflammatory effects on human lung cells, particularly upon inhalation exposure to CNC powders [156]. Furthermore, Yanamala et al. [157] discovered an inflammatory response in the lungs of mice after being exposed to wood-based CNC.

## Conclusion

The discovery of cellulose nanomaterial is perceived as a milestone in the field of materials science and engineering. This is proven by the global attention which cellulose nanomaterials have received in various applications, including bio-based food packaging, optoelectronics, and tissue regeneration, to name a few. The large surface area, environmentally friendly nature (low toxicity and renewable), and functionality (easily functionalized) of nanocellulose makes it a potential candidate for environmental applications such as wastewater and water treatment as well as carbon sequestration as demonstrated in this review article. Taking the time to understand the challenges linked to its preparation and its significance as catalyst, membrane, and treatment agents may help in its development for environmental remediation. Nanocellulose-based nanocomposites have demonstrated many advantages suited for environmental remediation in terms of heavy metal removal, dye removal, carbon sequestration, and antimicrobial activity. The main challenges lies with the energy consumption and yield during the preparation stage as well as the toxicity of its end product. Synergistic effects were observed in integrated treatment methods for the preparation of nanocellulose. On the other hand, modification of nanocellulose improved its functionality for the removal of a wide range of target pollutants. Despite its advantages, further investigation should aim to implement improved treatment schemes to effectively extract nanocellulose and its functionality as agents for environmental remediation in terms of economic viability and its performance in the treatment of actual wastewater.
